# IgG responses to the gSG6-P1 salivary peptide for evaluating human exposure to *Anopheles *bites in urban areas of Dakar region, Sénégal

**DOI:** 10.1186/1475-2875-11-72

**Published:** 2012-03-16

**Authors:** Papa M Drame, Vanessa Machault, Abdoulaye Diallo, Sylvie Cornélie, Anne Poinsignon, Richard Lalou, Mbacké Sembène, Stéphanie Dos Santos, Christophe Rogier, Frédéric Pagès, Jean-Yves Le Hesran, Franck Remoué

**Affiliations:** 1 Unité Mixte de Recherche MIVEGEC (IRD 224-CNRS 5290-UM1), Institut de Recherche pour le Développement, 34394, Montpellier Cedex 8, France; 2 Unité Mixte de Recherche 6236 (URMITE), Institut de Recherche Biomédicale des Armées, Allée du Médecin Colonel Jamot, 13262, Marseille Cedex 07, France; 3 Observatoire Midi-Pyrénées, Laboratoire d'Aérologie, Centre National de le Recherche Scientifique, Université Paul Sabatier, 31400, Toulouse, France; 4 Centre National d'Etudes Spatiales, Service Applications et Valorisation, 31401, Toulouse Cedex 9, France; 5 IRD/UMR 216-Mère et Enfant face aux Infections Tropicales, Faculté des Sciences Pharmaceutiques, 4 avenue de l'Observatoire, 75270, Paris Cedex 06, France; 6 Unité Mixte de Recherche 151, Institut de Recherche pour le Développement, Université de Provence, 13331, Marseille Cedex 03, France; 7 Département de Biologie Animale, Université Cheikh Anta DIOP, Dakar BP-5005, Sénégal; 8 Unité Mixte de Recherche 151, Institut de Recherche pour le Développement, Campus International de Recherche UCAD/IRD de Hann, Dakar BP 1386, Sénégal; 9 Institut Pasteur de Madagascar, B.P. 1274, Ambatofotsikely, Antananarivo 101, Madagascar; 10 Unité Mixte de Recherche MIVEGEC (IRD 224-CNRS 5290-UM1), Institut de Recherche pour le Développement, Centre de Recherche Entomologique de Cotonou (CREC), BP-4414, Cotonou, RP 01, Benin

## Abstract

**Background:**

Urban malaria can be a serious public health problem in Africa. Human-landing catches of mosquitoes, a standard entomological method to assess human exposure to malaria vector bites, can lack sensitivity in areas where exposure is low. A simple and highly sensitive tool could be a complementary indicator for evaluating malaria exposure in such epidemiological contexts. The human antibody response to the specific *Anopheles *gSG6-P1 salivary peptide have been described as an adequate tool biomarker for a reliable assessment of human exposure level to *Anopheles *bites. The aim of this study was to use this biomarker to evaluate the human exposure to *Anopheles *mosquito bites in urban settings of Dakar (Senegal), one of the largest cities in West Africa, where *Anopheles *biting rates and malaria transmission are supposed to be low.

**Methods:**

One cross-sectional study concerning 1,010 (505 households) children (n = 505) and adults (n = 505) living in 16 districts of downtown Dakar and its suburbs was performed from October to December 2008. The IgG responses to gSG6-P1 peptide have been assessed and compared to entomological data obtained in or near the same district.

**Results:**

Considerable individual variations in anti-gSG6-P1 IgG levels were observed between and within districts. In spite of this individual heterogeneity, the median level of specific IgG and the percentage of immune responders differed significantly between districts. A positive and significant association was observed between the exposure levels to *Anopheles gambiae *bites, estimated by classical entomological methods, and the median IgG levels or the percentage of immune responders measuring the contact between human populations and *Anopheles *mosquitoes. Interestingly, immunological parameters seemed to better discriminate the exposure level to *Anopheles *bites between different exposure groups of districts.

**Conclusions:**

Specific human IgG responses to gSG6-P1 peptide biomarker represent, at the population and individual levels, a credible new alternative tool to assess accurately the heterogeneity of exposure level to *Anopheles *bites and malaria risk in low urban transmission areas. The development of such biomarker tool would be particularly relevant for mapping and monitoring malaria risk and for measuring the efficiency of vector control strategies in these specific settings.

## Background

Currently, almost one billion people live in unstable or extremely low malaria risk areas, corresponding mainly to seasonal transmission, highlands (> 1,500 m), arid/semi-arid and urban areas [1]. In the latter, malaria can be a serious public health problem, especially in several rapid growing African cities where migrations of human populations from the countryside is intensive [2-4]. While urban development was generally believed to reduce the risk of malaria vector breeding, and thus malaria transmission, many African countries have declining economies, and most of them are struggling to cope with the pace and the extent of urbanization in their cities [2,5]. This may favour malaria vectors' breeding sites [6] and local malaria transmission [7]. In addition, people living in urban regions could be, in spite of their low exposure level to *Anopheles *vector bites, at a high risk of malarial morbidity and mortality, because of their delayed acquisition or lack of protective immunity [7]. The epidemiology of malaria in cities appeared therefore accurate, and urban malaria has been considered to be a major emerging problem of public health in Africa [8].

Dakar area is a typical example of sub-Saharan Africa's sprawling cities where malaria risk and transmission has been studied for several years. Malaria pathogens are transmitted by species of *Anopheles gambiae *complex (namely *An. gambiae s. l*.). *Anopheles arabiensis *is the most abundant *Anopheles *vector species and transmits mainly *Plasmodium falciparum *[9-12]. *Anopheles melas*, secondarily associated to *P. falciparum *transmission and *An. gambiae s. s*. M form have been described in low densities [10]. Malaria risk in Dakar is very focal, due to a high diversity in the degree and type of urbanization, the variation of density of human populations, the quality of water and waste management, the differential nature and use of vector control strategies and other own household factors [13]. A recent study has underlined possible changes in human exposure level to *Anopheles *bites in Dakar area. Indeed, due to an important increase of the building developments, estimated about 30% of the total area, the population at high risk for malaria fell from 32% to 20%, whereas the low risk population rose from 29 to 41% between 1996 and 2007 [14]. These findings suggest a current need to evaluate accurately the malaria risk in Dakar settings.

The evaluation of malaria risk using classical entomological methods presents considerable limitations in urban settings. Heavy sampling efforts are required to assess exposure level to *Anopheles *bites and then to evaluate the risk of malaria by entomological tools (trappings, human-landing catches, residual sprayings, etc.), especially in low urban exposure [15-17]. In addition, such methods are mainly applicable at population level and do not allow the evaluation of the heterogeneity of exposure between individuals. Moreover, these methods are not adapted to consider differential attractiveness to mosquitoes between individuals or other environmental and socio-economic factors, which could induce important variations in individual exposure to vector bites [17]. Such factors could be all the more considerable in urban areas. A simple, specific and highly sensitive indicator is therefore needed to evaluate the human exposure levels to *Anopheles *bites and potentially the risk of malaria in urban areas, at individual and population levels [18].

The measurement of antibody (Ab) response to vector saliva in human populations has been described to be a pertinent tool to assess the host exposure level to vector bites and the risk of vector-borne disease [19,20]. Salivary proteins of haematophagous arthropods facilitate blood feeding by counteracting haemostatic and inflammatory reactions and by modulating the immune response of the human or animal host [21,22]. Some of them can also induce a specific Ab response [23] which could represent a reliable indicator of vertebrate host exposure to vector-borne diseases in individuals bitten by arthropod vectors, such as ticks [24], sand-flies [25], *Triatoma *[26], *Glossina *[27], *Aedes *[28,29], *Culex *[30], *An. gambiae *[31,32], *Anopheles dirus *[33] and *Anopheles darlingi *[34]. But, the use of whole saliva could not give a pertinent biomarker, because of i) the potential cross-reactivity with salivary epitopes of other haematophagous arthropods; ii) the lack of reproducibility between saliva batches, and iii) an inadequate production needed for large-scale studies [35,36]. For these purposes, a specific, antigenic, easy synthesized and highly conserved peptide between *Anopheles *mosquitoes, the gSG6-P1 (*An. gambiae *Salivary Gland Protein-6 peptide 1), has been identified and validated as a more pertinent biomarker of *Anopheles *bites [36]. Indeed, specific IgG responses to this gSG6-P1 peptide seemed to give an accurate evaluation of low and very low-level exposure to *An. gambiae *[18] as well as to *Anopheles funestus *bites, the second major malaria vector in Africa [37]. Recently, it has been also shown that Ab response to gSG6-P1 peptide offers a useful biomarker for a reliable assessment of the efficacy of impregnated bed-net use against exposure to *Anopheles *bites [35]. In addition, specific IgG response to gSG6-P1 does not seem to build up but wanes rapidly, when exposure failed. This property represents a major strength for its use for evaluating the human exposure to mosquito bites in low-exposure contexts [35].

The aim of the present study was to evaluate the exposure level to malaria vectors in individuals living in a supposed low endemic urban area (Dakar) by using specific human IgG responses to the *Anopheles *gSG6-P1 salivary peptide biomarker.

## Methods

### Study site

**Figure 1:**
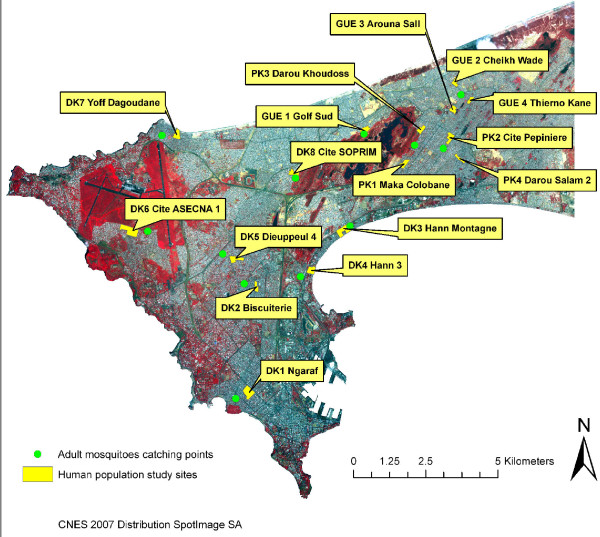
**Localization of the studied sites in Dakar**. The 12 adult mosquito-catching (in green) and the 16 blood spot-sampling (in yellow) sites are localized on the map. An adult mosquito catching point allowed an estimate of *An. gambiae *intensity of exposure in two (PK 2 and PK 4) or three (GUE 1, GUE 2 and GUE 3) neighbouring blood spot-sampling sites. DK, PK and GUE are respectively Dakar, Pikine and Guediawaye, departments of Dakar region. The brown base of the map represents the area without human habitations. Its darkness is correlated to the presence of vegetation.

The region of Dakar (the capital city of Senegal) is located in the Cape Verde Peninsula (14° 43' 29.06" North, 17° 28' 24.06" West) at the western point of Africa. In 2008, Dakar population was estimated to 2,500,000 inhabitants, amounting to 21% of the total population of the country (about 12,000,000 inhabitants), with a high population density (4,459 inhabitants/km^2^). Globally, the studied region is a coastal plain area and has a mild sahelian climate with a hot and wet season which lasts from June to November and is characterized by average temperatures between 24 and 30°C. In 2008, the annual average rainfall was 510 mm and peaked in August and September [38]. The study was conducted in 2008, from the end of the rainy season (October) to the beginning of the dry season (December), in different areas of downtown Dakar (Dakar department) and suburbs (Pikine and Guediawaye departments) (Figure 1). *Plasmodium *species (mainly *P. falciparum*) are transmitted by *An. gambiae s.l*. mosquitoes (mainly *An. arabiensis*) and malaria transmission occurs from July to December, with a peak from September to November [9,10,13].

### Study design

Dakar region comprises 42 urban districts (UDs), which are constituted by about 2,000 census districts (CDs), the smallest level in terms of demographic inventory in Senegal. This work began by mapping individual and household information (socio-cultural and demographic characteristics, economic level) stemming from the general inventory of the Senegalese population and housing of 2002. An analysis of the main socio-demographic and economic variables, using principal component analysis and the k-means clustering method, was performed and allowed to classify CDs in five types. One of the most representative types in each UD was randomly chosen; 42 CDs were then selected. To reach the number of 50 sites planned for the study, eight other CDs were added according to their socio-demographic characteristics and their proximity to shallows in order to get more information on districts with the highest risk of malaria. Selected CDs were visited by one of the five teams of investigators according to itineraries pre-established by the head-supervisor; 60 households in each CDs (3,000 households for the 50 sites) were selected and identified. The first criterion of a household selection was the presence of a two to 10 years-old child resident. After having the written agreement of resident family, investigators crossed a questionnaire about the household lifestyle, income and the access mode to healthcare facilities. Concomitantly, the adult woman (generally the child's mother) who answered the questionnaire was selected for blood sampling. Pregnant women, individuals who were sick and/or have taken anti-malarial drugs during the last 15-30 days preceded nurses' passage were not included in the cohort.

In total, due to refusal or exclusion criteria, 4,658 individuals (2,231 children and 2,427 adults, women in majority) from 50 sites of Dakar region were included in the study. A dried blood spot for immunological analysis were collected for each individual. Results are presented here for a sub-sample of 1,010 individuals (505 children two to 10 years old and 505 women > 18 years (adults)) randomly sampled within 16 CDs for which entomological data are available (Figure 1). The number of individuals (children and women) for whom IgG Ab responses were assessed varied by site, from 56 in DK 1 to 76 in DK 3 (Table 1).

**Table 1:** Blood sampling and entomological survey periods

Districts name	Districts code	Blood sampling dates	n	Benning of mosquitoes sampling	Number of sampling nights*
Ngaraf	DK 1	Dec-08	56	Aug-08	10
Biscuiterie	DK 2	Dec-08	60	Aug-08	9
Hann Montagne	DK 3	Oct-08	76	Jul-08	6
Hann 3	DK 4	Oct-08	64	Jul-08	6
Dieupeul 4	DK 5	Nov-08	58	Aug-08	7
Cité ASECNA 1	DK 6	Oct-08	70	Aug-08	5
Yoff Dagoudane	DK 7	Nov-08	62	Aug-08	8
Cite SOPRIM	DK 8	Oct-08	58	Jul-08	7
Golf Sud	GUE 1	Nov-08	64	Aug-08	9
Cheikh Wade	GUE 2	Nov-08	60	Aug-08	8
Arouna Sall	GUE 3	Nov-08	62	Aug-08	9
Thierno Kane	GUE 4	Nov-08	70	Aug-08	8
Maka Colobane	PK 1	Nov-08	60	Jul-08	8
Cité Pépinière	PK 2	Nov-08	62	Jul-08	9
Darou Khoudoss	PK 3	Nov-08	58	Jul-08	9
Darou Salam 2	PK 4	Nov-08	70	Jul-08	8

DK (= Dakar), GUE (Guédiawaye) and PK (Pikine) are initials of the names of the department of the studied district. "n" represents the number of individuals blood-sampled for immunological assays in each district. The number of sampling-nights is reported for each district from the first entomological survey day (during the beginning of the transmission period) to the beginning of blood spot samplings in the concerning district

In the immunological sub-sample, the median age was 5.0 (Q25% = 3.0 and Q75% = 8.0) in children and 35.0 (Q25% = 28.0 and Q75% = 42.0) years old in adult women of all districts. The median age was similar between districts, except in women where it was significantly different only between PK 4 and DK 7 and between PK 4 and PK 1 (p < 0.05). This study was conducted in accordance with the Edinburgh revision of the Helsinki Declaration, and was approved by the ethical committees of the Ministry of Health and Prevention of Senegal (December 2008). Written informed consent was obtained for all individuals enrolled in the study. For children, this informed consent was signed by one of their parents or their tutor (child guardian).

### Entomological analysis

Adult mosquito samplings were carried out once every two weeks during the study period in 12 study sites located in downtown Dakar and its closed suburbs (Pikine and Guédiawaye) from July 2008 to the date of blood-spot samplings (October to December 2008 according to CDs). Human-landing catches of adult mosquitoes were conducted both indoor (one catching point) and outdoor (two catching points) in or near (less than 250-300 m from the epicentre of the CD) each CD. Five to 10 study nights were carried out according to CDs. Indoor captures were conducted with the window or door slightly ajar. Collectors gave prior informed consent and received yellow fever vaccination and anti-malarial chemoprophylaxis consisting of 100 mg doxycycline per day for the duration of the study and one month after. Two collectors were contracted for each catching point to work from 8:00 p.m. to 7:00 a.m., with each one resting every two hours. Collectors were rotated among the catching points on different collection nights to minimize sampling bias. The mosquitoes were recorded by catching point, date and hour of capture and they were sorted by genera. The Anopheline mosquitoes were identified morphologically following the Gillies and Coetzee keys [39]. The human biting rate (HBR) was expressed as the number of female *An. gambiae s.l*. per person per night, averaged for both outdoor and indoor catching points. Thus, the HBR for each district accounted for the average cumulative bites per person per night received before the blood sampling.

### Salivary peptide gSG6-P1

The gSG6-P1 peptide was designed using bioinformatics to maximize its *Anopheles *specificity and its antigenicity, as previously described [36]. The gSG6-P1 peptide was synthesized, purified (> 95%) by Genepep SA (St-Clément de Rivière, France). All peptide batches were shipped in lyophilized form and then suspended in 0.22 μm ultra-filtered water and frozen in aliquots at 80°C until use for immunological tests (ELISA).

### Evaluation of human IgG antibody levels (ELISA)

Standardized dried blood spots (80 mm diameter) were eluted as previously described [31]. ELISAs were carried out on dried blood-spot eluates to assay IgG response to the gSG6-P1 antigen as previously described [35]. Individual results were expressed as the ΔOD value: ΔOD = ODxODn, where ODx represents the mean of individual optical density (OD) in both antigen wells and ODn the individual OD in a blank well containing no gSG6-P1 antigen. Specific anti-gSG6-P1 IgG levels were also assayed in non-*Anopheles *exposed individuals (n = 14-neg; North of France) in order to quantify the non-specific background Ab level and to calculate the cut-off of immune response (threshold of immune response or TR = mean (ΔDO_neg_) + 3SD = 0.204). An exposed individual was then classified as an immune responder if its ΔOD > 0.204.

### Statistical analysis

All data were analysed with GraphPad Prism5 software^® ^(San Diego, CA, USA). The non-parametric Mann-Whitney *U*-test was used for comparison of median Ab levels between children and adult women of each district, the non-parametric Kruskal-Wallis test for comparison of medians for more than two groups and the Fischer exact test for comparison between two proportions. A simple linear regression was used to evaluate association level between IgG responses and exposure levels to *An. gambiae s.l*. bites (HBR). Statistical significant difference between the three defined groups of exposure has been assessed using an ANOVA simple test. All differences were considered significant at P < 0.05.

## Results

### Specific IgG responses to gSG6-P1 peptide according to districts

**Figure 2:**
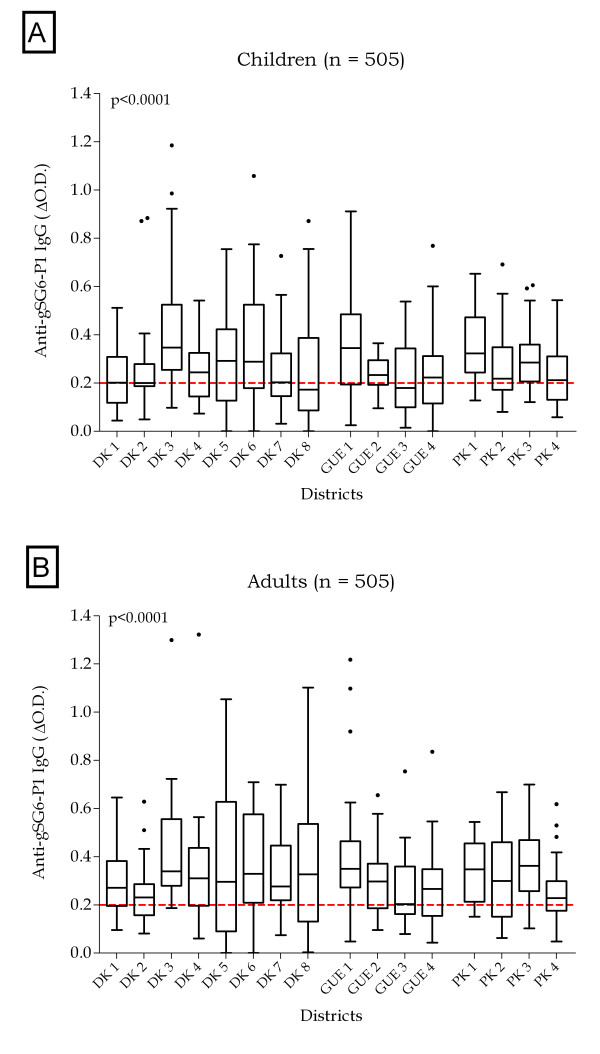
**Children and adult women IgG antibody levels to gSG6-P1 in the 16 studied sites**. Individual IgG responses (ΔOD) to gSG6-P1 peptide are presented and bars indicate the median value for studied individuals in each district. The boxes locate the middle 50% of the data; horizontal lines in the boxes indicate medians of the data; lengths of boxes correspond to the inter-quartile ranges. The horizontal red dotted line represents the cut-off of IgG responder. Statistical significant differences of specific IgG between districts are indicated (P < 0.0001; non-parametric Kruskal-Wallis test).

Specific IgG Ab levels to gSG6-P1 peptide were evaluated according to studied sites (CDs) and presented for children (Figure 2A) and adult women (Figure 2B). Results of this analysis show considerable variations within and between studied CDs. In spite of the inter-individual heterogeneity at CD level, the median of specific IgG Ab levels in children as well as in adults differed significantly (P < 0.0001) between CDs. Moreover, CDs presenting high or low specific IgG levels in children were the same where high or low IgG Ab levels were respectively observed in adult women. The median of specific IgG Ab levels was particularly high in DK 3 and DK 6, in GUE 1 and in PK 1 and PK 3 and low in DK1, DK 2, DK 7 and DK 8 (four populous districts of Dakar), in GUE 3 and in PK 2 and PK 4 (suburbs of Dakar region). In addition, in the majority of districts (13/16), the median of anti-gSG6-P1 IgG levels seemed to be higher in adult women (Figure 2B) than in children (Figure 2A) but this difference was not significant, except in DK 8 (P = 0.01).

As observed for median of specific IgG Ab levels percentages of individual immune responders (individuals with ΔOD > 0.204 = TR) were fairly high (minimum: 43.55% in GUE 3 and maximum: 86.84% in DK 3) and varied according to CDs (Table 2). Indeed, the lowest percentage of responders were observed in GUE 3 (43.55%), DK 1 (50.00%), DK 2 (50.00%), PK 4 (54.29%) and DK 7 (54.84%) and the highest in DK 6 (74.29%), GUE 1 (81.25%), PK 3 (82.76%), PK 1 (83.33%) and DK 3 (86.84%). The use of median IgG level or the percentage of immune responders appeared, therefore, to give similar results for the classification of CDs as high or low anti-gSG6-P1 IgG response.

**Table 2:** Constitution of exposure groups according to percentages of immune responders and median anti-gSG6-P1 IgG responses

"Exposure" groups	Districts code	% of anti-gSG6-P1 responders	Rank 1	Median of anti-gSG6-P1 IgG	Rank 2	Rank sum	*An. gambiae *Bites/Human/Nigh
Group 1	GUE 3	43.55	1	0.189	1	2	6.6
	DK 2	50,00	3	0.209	2	5	0.3
	DK 1	50,00	2	0.234	4	6	0.8
	PK 4	54.29	4	0.213	3	7	14.3
Group 2	DK 7	54.84	5	0.262	7	12	0.4
	GUE 4	61.43	7	0.253	5	12	7.4
	PK 2	59.68	6	0.264	9	15	14.3
	DK 4	64.06	9	0.262	8	17	17.1
	GUE 2	70,00	11	0.255	6	17	7.4
	DK 5	62.07	8	0.292	11	19	1.5
	DK 8	65.52	10	0.273	10	20	46.0
Group 3	DK 6	74.29	12	0.328	14	26	26.2
	PK 3	82.76	14	0.316	12	26	33.9
	GUE 1	81.25	13	0.350	15	28	121.4
	PK 1	83.33	15	0.323	13	28	37.0
	DK 3	86.84	16	0.350	15	31	82.9

Districts are classed in an ascending order according to their proportion of anti-gSG6-P1 IgG immune responders (Rank 1 column) and the median of specific IgG level (Rank 2 column). A rank number was assigned to each district according its position in the considering rank column. The sum of the two rank numbers of each district was realized

### Specific IgG responses and intensities of exposure to *Anopheles gambiae sensu lato *bites

**Figure 3:**
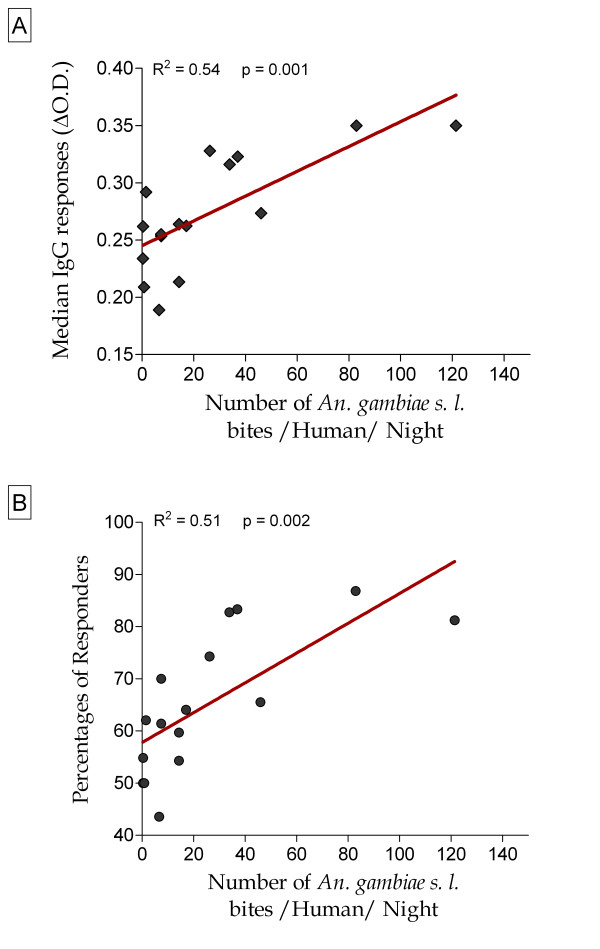
**IgG response to gSG6-P1 and intensity of exposure to *Anopheles gambiae sensu lato *according to the studied sites**. The medians of anti-gSG6-P1 IgG levels (Figure 3A) and the percentages of immune responders (Figure 3B) for all individuals (children and adults) are correlated to the number of *An. gambiae *bites/human/night according to districts. Black rhombus and black circles represent respectively median anti-sGS6-P1 IgG levels and percentage of human responders in the 16 CDs. The red solid line indicates the correlation line between each immunological parameter and the *An. gambiae s. l*. human biting rate. Statistical significant linear associations are indicated (simple linear regression method).

Correlations between immunological parameters, i.e. the human IgG level (Figure 3A) and the percentage of immune responders (Figure 3B) to gSG6-P1 peptide, and entomological data, the number of *An. gambiae s.l *bites/human/night (HBR), are presented for all studied population (children and adult women).

The entomological data (Table 2) indicate that the intensity of exposure *An. gambiae s.l*. bites was globally low and changed also according to CDs (HBR = 0.3 in DK 1 to 121.4 in GUE 1). It was positively and significantly correlated to medians of specific IgG levels (R^2 ^= 0.54; Figure 3A) and to the percentage of immune responders (R^2 ^= 0.51; Figure 3B). Similar correlation trends were also observed when specific IgG data are separately analysed for children and adult women. Indeed, exposure levels to *An. gambiae s.l *were positively associated to anti-gSG6-P1 IgG levels and to the percentage of anti-gSG6-P1 IgG responders in children (R^2 ^= 0.54 with p = 0.001 and R^2 ^= 0.33 with p = 0.02, respectively) and in adult women (R^2 ^= 0.30 with p = 0.03 and R^2 ^= 0.59 with p = 0.0005, respectively) (graphs not shown). Interestingly, in the studied districts, *An. gambiae s. l*. HBR appeared to be highly correlated to medians of specific IgG levels in adult women, whereas they were more clearly correlated to percentages of immune responders in children.

However, results show some discrepancies between immunological and entomological results in some CDs. Indeed, low specific IgG levels and/or percentage of responders could be observed in CD where high HBR were detected and vice versa (Table 2). For example, GUE 3 (HBR = 6.6) presented low specific IgG level and/or percentage of responders than DK 1 (HBR = 0.3), DK 2 (HBR = 0.8), DK 5 (HBR = 1.5) and DK 7 (HBR = 0.4). In contrast, PK 3 (HBR = 33.7) presented a higher percentage of responders than DK 8 (HBR = 46.0) and GUE 1 (HBR = 121.4).

### IgG response to gSG6-P1 peptide as an indicator for a reliable evaluation of urban exposure level to *Anopheles gambiae s.l*. bites

**Figure 4:**
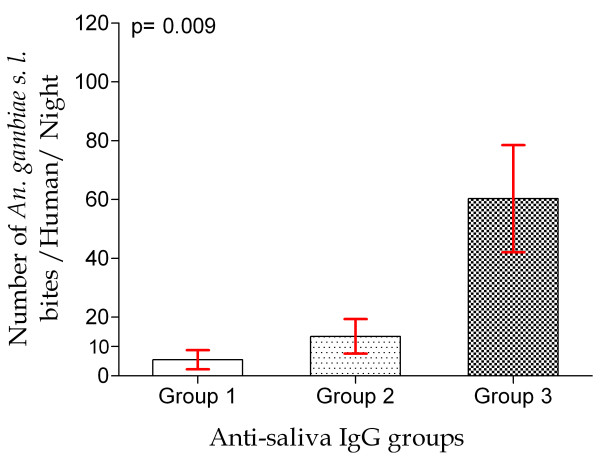
**Intensity of exposure to *Anopheles gambiae sensu lato *according to the three immunological exposure groups**. Data present the mean of the number of *An. gambiae *bites/human/night according to the three defined exposure groups. Statistical significant difference between the three groups is indicated using an ANOVA simple test and differences between each pair of groups by the Bonferroni's Multiple Comparison Test.

The first step was to classify the districts within three groups (gr. 1 = low; gr. 2 = medium and gr. 3 = high level), based to the percentage of immune responders and the medians of specific IgG responses, as described in Table 2. As expected, the percentage of immune responders and the specific IgG level increased according the immunological groups and were highly different between each pair of groups (p < 0.0001). The intensity of exposure levels to *An. gambiae *bites (HBR) evaluated by classical entomological methods was then compared between the three groups of immune response (Figure 4). It increased according to the groups of immune response. However, this difference was only clear when taking into account the group presenting the highest level of specific immune response (group 3). Indeed, the intensity of exposure levels (HBR), showed significant differences only in comparing group 3 versus group 1 and group 3 versus group 2. No significant difference was observed between group 1 and group 2 (Figure 4).

## Discussion

The present study focused on the application of the *Anopheles *gSG6-P1 salivary biomarker in the evaluation of the exposure level to vector bites in low-urban malaria settings. Firstly, data of the study have shown a high heterogeneity of specific IgG Ab response to this salivary peptide between individuals, i) within each studied district, and ii) between the 16 studied districts of Dakar and its suburbs. These observations suggest in this urban area important differences in the human-*Anopheles *contact level which could be influenced by several individual and/or household factors and behaviours [40]. For example, the use of vector control strategies [41] and movements of urban populations to rural areas, where exposure to *Anopheles *bites is higher [7], can increase/decrease significantly the probability of contact between human hosts and malaria vectors and explain a part of observed immunological results. But some epidemiological factors such as genetic, co-infection, nutritional parameters, could not be excluded. Nevertheless, these results strengthen previous clear data that validated this salivary peptide of *An. gambiae *as a relevant tool biomarker to assess the human exposure level to malaria vectors bites in other exposure and malaria transmission settings [18,35-37].

Secondly, results have shown that immunological data were positively correlated to *An. gambiae s.l*. human biting rates (HBR). This result suggests that differences in exposure levels to *An. gambiae *bites in the Dakar area could partly explain the variations in anti-gSG6-P1 IgG Ab response observed between districts and groups of exposure. Similar observations were reported in previous studies carried out in individuals exposed to *An. gambiae *bites in a rural area of Senegal [18] and semi-urban area of Angola. However, in urban settings, differences in exposure level to *Anopheles *bites between neighbouring districts can be generated by local differences in larval breeding sites which are mostly localized and advantaged or disadvantaged by many environmental modifications [13]. They could be also linked to the low dispersion capacity of *An. gambiae s. l*. populations in urban areas (less than 300 m from their breeding sites) due to the high density of human populations [14] and their high availability as hosts for *Anopheles *bites [14,42]. In the Dakar area, recent studies have reported the existence of farming and gardening in some districts of Hann-Yarakh (including DK 3 and DK 4), Golf Sud (GUE 1) and Pikine (PK 1 and PK 3) areas [11,13] where high levels of specific anti-gSG6-P1 IgG responses were observed. But, irrigation practices seemed here to be extremely rare or inexistent, in contrast to observations reported in urban cities of Ivory Coast [43] and Ghana [44].

However, discrepancies observed in the correlation between immunological parameters and the exposure level to *An. gambiae *bites in some CDs (GUE 3 for example) suggest that other factors may contribute to explain the variation in individual IgG responses to gSG6-P1 peptide, and then human-*Anopheles *mosquitoes contact. For example, a differential use of vector control tools (ITNs, sprays, curtains) can reduce drastically human-vector contact. Many household characteristics (height, type, use of air conditioning, well-closed windows), which can differ between districts, could also be crucial factors. This study highlights then the pertinence of the use of this peptide biomarker for evaluating the individual and population exposure to bites and the efficacy of vector control devices as it has been demonstrated in a semi-urban area [35].

Thirdly, it has observed that the means of exposure intensity to *An. gambiae *bites were significantly different only between the high immune response group and the two other groups (medium and low level). This observation deals with the usefulness of such biomarker in the evaluation of exposure levels to bites, especially in low/very low exposure contexts (for example immune response groups 1 and 2), where current entomological methods can give inaccurate estimations of the human-mosquito contact as also previously observed [18]. However, the present entomological data must be interpreted with cautious. Indeed, mosquitoes' catch sites were sometimes more than 250-300 m distant from the epicentre of human blood sampling site and might not reliably evaluate *An. gambiae s.l*. HBR in concerned areas.

The gSG6-P1 peptide was designed on the basis of the *An. gambiae s.s*. sequence, the only *Anopheles *genome completely available [45]. In the studied area, species of *An. gambiae *complex, especially *An. arabiensis *and *An. melas*, are the main vectors of *P. falciparum *[10]. *Anopheles pharoensis *has been also described in few quantities, but was not associated to *Plasmodium *transmission [13]. It can be hypothesized that the exposure of individuals to these *Anopheles *anthropophagic vectors has been evaluated using this salivary peptide, which is highly conserved between *Anopheles *species [36,46]. Indeed, it has been shown that gSG6-P1 peptide shares 82% and 91% identity with *Anopheles stephensi *and *An. funestus *and that IgG response to this peptide was also biomarker of *An. funestus *exposure [37]. All these data tend to support the proposal that gSG6-P1 can be used reliably to evaluate children and adults low-exposure levels to major *Anopheles *species known to be vector of malaria in sprawling African cities, and then to assess malaria risk in such areas.

## Conclusions

To identify new biomarkers for malaria risks, an efficient tool based on the measurement of human IgG Ab responses to specific gSG6-P1 peptide represents a new way to evaluate accurately the heterogeneity of exposure level to bites at population and at individual levels. This approach appears to be promising and complementary to classical entomological methods, because it can give a reliable evaluation of the individual contact with anthropophilic *Anopheles *in particular low exposure urban areas. This biomarker could be particularly relevant in mapping malaria risk in urban settings, but also in high -altitude or seasonal malaria, and in travellers in endemic areas. It could also represent an alternative way to obtain new criteria for measuring the efficacy of the specific vector control strategies used in urban settings and allow then a pertinent monitoring of strategies developed by the National Malaria Control Programmes in African urban contexts.

## Competing interests

The authors declare that they have no competing interests.

## Authors' contributions

PMD and VM participated in field surveys, assembled and analysed data and drafted the manuscript. AD participated in study coordination, field surveys and data collection. SC and AP participated in manuscript preparation, writing and revision. SD and RL coordinated the study and revised the manuscript. MS helped with manuscript preparation and revised the manuscript. JYH (participated also to the study coordination); CR and FP designed the study, helped with manuscript preparation and revised the manuscript. FR participated in study design, preparation and writing of the manuscript and revised the manuscript. All authors read and approved the final manuscript.
